# The future impact of population growth and aging on coronary heart disease in China: projections from the Coronary Heart Disease Policy Model-China

**DOI:** 10.1186/1471-2458-8-394

**Published:** 2008-11-27

**Authors:** Andrew Moran, Dong Zhao, Dongfeng Gu, Pamela Coxson, Chung-Shiuan Chen, Jun Cheng, Jing Liu, Jiang He, Lee Goldman

**Affiliations:** 1 Division of General Internal Medicine, Columbia University Medical Center, New York, USA; 2 Columbia University College of Physicians and Surgeons, New York, USA; 3 Department of Epidemiology, Beijing Institute of Heart, Lung, & Blood Vessel Diseases, Beijing, PR China; 4 Capital University of Medical Sciences, Beijing, PR China; 5 Department of Evidence Based Medicine, Cardiovascular Institute and Fu Wai Hospital of the Chinese Academy of Medical Sciences, Beijing, PR China; 6 National Center for Cardiovascular Diseases, Beijing, PR China; 7 University of California at San Francisco, San Francisco, USA; 8 Department of Epidemiology, Tulane University School of Public Health and Tropical Medicine, New Orleans, USA; 9 Department of Medicine, Tulane University School of Medicine, New Orleans, USA

## Abstract

**Background:**

China will experience an overall growth and aging of its adult population in coming decades. We used a computer model to forecast the future impact of these demographic changes on coronary heart disease (CHD) in China.

**Methods:**

The CHD Policy Model is a validated state-transition, computer simulation of CHD on a national scale. China-specific CHD risk factor, incidence, case-fatality, and prevalence data were incorporated, and a CHD prediction model was generated from a Chinese cohort study and calibrated to age-specific Chinese mortality rates. Disability-adjusted life years (DALYs) due to CHD were calculated using standard methods. The projected population of China aged 35–84 years was entered, and CHD events, deaths, and DALYs were simulated over 2000–2029. CHD risk factors other than age and case-fatality were held at year 2000 levels. Sensitivity analyses tested uncertainty regarding CHD mortality coding, the proportion of total deaths attributable to CHD, and case-fatality.

**Results:**

We predicted 7.8 million excess CHD events (a 69% increase) and 3.4 million excess CHD deaths (a 64% increase) in the decade 2020–2029 compared with 2000–2009. For 2030, we predicted 71% of almost one million annual CHD deaths will occur in persons ≥65 years old, while 67% of the growing annual burden of CHD death and disability will weigh on adults <65 years old. Substituting alternate CHD mortality assumptions led to 17–20% more predicted CHD deaths over 2000–2029, though the pattern of increases in CHD events and deaths over time remained.

**Conclusion:**

We forecast that absolute numbers of CHD events and deaths will increase dramatically in China over 2010–2029, due to a growing and aging population alone. Recent data suggest CHD risk factor levels are increasing, so our projections may underestimate the extent of the potential CHD epidemic in China.

## Background

**Figure 1:**
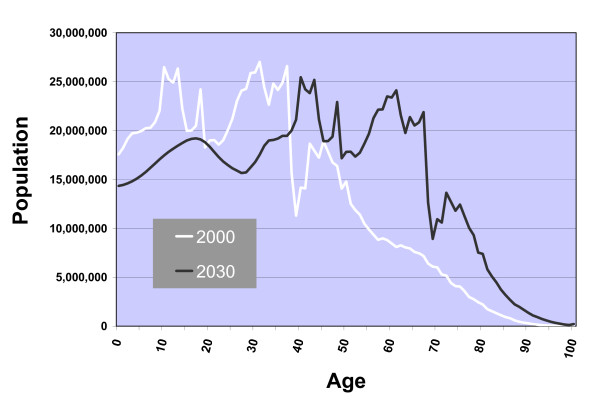
**Age distribution of the Chinese population in 2000 and 2030.** Distributions are based on U.S. Census Bureau International Database projections.

China, the most populous of nations, will have a growing and aging population in coming decades. China's population was 1.27 billion in 2000, with seven percent of the population ≥ 65 years old; by 2030 the population is predicted to be 1.46 billion, and 16% percent of Chinese citizens will be ≥ 65 (Figure 1).[1,2] The aging of the population is due both to a boom in births in China during the 1950's to early 1970's and to an increased life expectancy for many Chinese.[3] The latter phenomenon has been attributed to the "epidemiologic transition" – a shift away from illnesses that cause mortality in the young (e.g., infectious and maternal/fetal diseases) toward chronic diseases that affect the old. [4-8]

Cardiovascular disease is already the leading cause of mortality in China, and coronary heart disease (CHD) is the 4^th ^leading cause. [8-10] Past analyses have estimated the future societal consequences of cardiovascular disease in China,[9] and analyzed the increasing CHD mortality trend in Beijing.[11] We sought to quantify the extent to which population growth and aging alone would impact the epidemic of CHD in China in coming decades in greater detail and on a national scale. We built upon the CHD Policy Model, a computer predictive model that was initially developed for the United States,[12] adapted the model for use in China, and forecasted CHD events and disability-adjusted life years (DALYs) in Chinese adults from 2000–2029. In this baseline analysis, levels of CHD risk factors other than age [i.e., blood pressure (BP), cholesterol, body mass index (BMI), diabetes, and smoking] were held constant over time. We also conducted sensitivity analyses in order to examine the impact of future demographic changes on CHD in China under variable cause-specific mortality and CHD case-fatality assumptions.

## Methods

### The Coronary Heart Disease Policy Model

The CHD Policy Model is a computer-simulation, state-transition (Markov cohort) model that has been used for 20 years to predict CHD incidence, prevalence, mortality, and costs in U.S. adults aged 35–84. [12-22] The CHD Policy Model-China is comprised of three submodels: the demographic-epidemiologic submodel, the bridge submodel, and the disease history submodel. The demographic-epidemiologic submodel predicts CHD incidence and non-CHD mortality among the population without CHD, stratified by age, sex, and up to six additional categorized risk factors: systolic blood pressure (BP, <120, 120–139, ≥140 mmHg), smoking status (active smoker, non-smoker with exposure to environmental tobacco smoke, non-smoker without environmental exposure), high density lipoprotein (HDL) cholesterol [<0.9, 0.9–1.3, or >1.3 mmol/L (<35, 35–49, >49 mg/dL)], low-density lipoprotein (LDL) cholesterol [<2.6, 2.6–3.4, or >3.4 mmol/L (<100, 100–130, >130 mg/dL)], BMI (<25, 25–29, >29 kg/m^2^), and diabetes mellitus (yes or no). After CHD develops, the bridge submodel characterizes the initial CHD event and its sequelae for 30 days. Then, the disease history submodel predicts subsequent CHD events, revascularization procedures, CHD mortality, and non-CHD mortality among patients with CHD. All population distributions, risk factor levels, coefficients, event rates, and case fatality rates can be modified for forecasting simulations. Additional methods for the Policy Model-China are provided in Additional file 1. A general description of the United States version of the CHD Policy Model software is available elsewhere.[23]

### Overview of China-specific model inputs

The current analysis introduces the CHD Policy Model-China, a model modified and calibrated to predict CHD in the Chinese population. We employed China-specific data inputs from population-based studies wherever possible, including age- and sex-specific estimates of the Chinese population distribution during 2000–2029, as well as CHD incidence, prevalence, case-fatality, and mortality, non-CHD mortality, and rates of coronary revascularization (Table 1).

**Table 1:** Primary model inputs and references for the coronary heart disease (CHD) Policy Model-China

**Variable**	**Source**
Population of China 2000–2029*	United States Census Bureau International Database,[1] Chinese National Bureau of Statistics[2]
Incidence of CHD	China Multi-provincial Cohort Study (CMCS),[27] 1992–2002 Sino-MONICA,[24] Beijing, 1993–2004
Prevalence of CHD in 2000	International Collaborative Study of Cardiovascular Disease in Asia Study (InterASIA),[28] 2000–2001 China National Hypertension Survey Epidemiology Follow-up Study (CHEFS),[8] 1991–2000
Total and Cause-Specific Mortality	
Total	For total mortality: Global Burden of Disease Study, 2002 [36]
CHD	For cause-specific mortality:
Non-CHD	CHEFS[8], Global Burden of Disease Study, 2002 [36]
CHD risk factor means and joint distributions, 2000	InterASIA [28,52-55,59]
Risk factor hazards for CHD	CMCS [27]
One-day and 28-day CHD case-fatality	Sino-MONICA, Beijing 1993–2004 [24]
Revascularization rates	Bridging the Gap in Coronary Heart Disease Secondary Prevention (BRIG) Study 2006-present (unpublished data, provided by personal communication, Dong Zhao, M.D., Ph.D., March, 2007)
Disability Adjusted Life Years (DALY) disability weights and assumptions	Global Burden of Disease Study, 2001[35]

*Sino-MONICA*, conducted from 1985–1993 in 17 monitoring units in 16 provinces of China, was a surveillance sample of roughly 5,000,000 people, and Sino-MONICA surveillance has continued from 1994 to the present in a sub-population of approximately 170,000 people in Beijing. [24-26] All acute coronary heart disease and stroke events in persons 25–74 years old were registered using the World Health Organization (WHO) Monitoring Trends and Determinants in Cardiovascular Disease (MONICA) project methodology and criteria.[24] The *Chinese Multi-provincial Cohort Study (CMCS)*.[27] recruited 27,003 participants from 11 provinces of China in 1992–1993. An additional 3,118 participants were added in 1996 and 1999. Prevalent cases of CHD were excluded at baseline, and baseline variables measured. The cohort was followed for 10 years for CHD and non-CHD events and deaths, using MONICA case finding methods and criteria.[24]

The *International Collaborative Study of Cardiovascular Disease in Asia Study (InterASIA)*.[28] enrolled a nationally-representative multistage cluster sample of 15,838 Chinese adults, aged 35–74 years in 2000–2001. Fasting glucose, BP, BMI, and LDL and HDL cholesterol were measured. Demographic, medical history, and tobacco exposure information was collected in interviews. The *China National Hypertension Survey Epidemiology Follow-up Study (CHEFS) *[8] used a multi-stage, random clustering design to identify a nationally-representative sample of 83,533 men and 86,338 women older than age 40 who were eligible for follow-up beginning in 1991. Follow-up mortality data were gathered from 158,666 participants or proxies by interview in 1999 (93.4% follow-up rate).

The *Bridging the Gap in CHD secondary prevention (BRIG) Study *is an ongoing longitudinal survey of Chinese patients diagnosed with CHD and aims to measure adherence to evidence-based inpatient and outpatient CHD management practices representative of all of China. BRIG sampled 64 hospitals (32 tertiary and 32 secondary-level hospitals) located in 22 provinces, five autonomous regions, and four municipalities located in urban and rural areas throughout China (Personal communication, Jun Cheng, MD, September, 2008). At the time of this analysis, the BRIG study had recruited 3,174 participants hospitalized for an acute CHD event (48.7% acute myocardial infarction, 45.6% unstable angina, 5.4% ill-defined CHD). Information was collected regarding patients' demographic characteristics, medical history, presenting symptoms, electrocardiographic findings, biochemical examinations, cardiac biomarker assays, clinical characteristics, use of cardiac medications, and revascularization procedures, including percutaneous coronary intervention (PCI), coronary artery bypass graft surgery (CABG), and thrombolysis. Overall rates of revascularization for acute myocardial infarction in the BRIG study were 31% in men and 17% in women. All costs and hospital-associated and outpatient outcomes were recorded, including outcomes of interventional procedures.

For most population-based studies in China, data were available only for Chinese adults aged 35–74 years. We therefore applied age-related trends in CHD incidence from the Framingham Heart Study[29] and Olmsted County, Minnesota, U.S.A.[30] and age-related trends in case-fatality rates from the MONICA Study[31] and U.S. National Hospital Discharge Survey and California Office of Statewide Health Planning and Development hospitalization records[23] to estimate these parameters for the 75–84 year old category. Annual transition rates in risk factor levels were calculated to preserve age-related risk factor trends observed in the InterASIA survey in 2000–2001 using methods developed for the United States CHD Policy Model.[23]

### Population Assumptions

The estimated population of China aged 35–84 years in thee year 2000, by age and sex, was entered from the United States Census Bureau International Database, because that database provided both the Chinese population in 2000 and projections of the population between the years 2001–2029.[1] Year 2000 estimates from this database were compared with estimates from the National Bureau of Statistics, China, and found to be similar for this age range.[32] Numbers of 35 year old adults entering the model from the year 2001 to the year 2030 were also entered from U.S. Census Bureau International Database projections.

### Mortality Assumptions

The CHD Policy Model-China defined CHD as myocardial infarction (ICD-9 410, 412 or ICD-10 I21, I22), angina and other CHD (ICD-9 411, 413 and 414, or IC-10 I20, I23–I25), and a fixed proportion of "ill-defined" cardiovascular disease coded events and deaths (ICD-9 codes 427.1, 427.4, 427.5, 428, 429.0, 429.1, 429.2, 429.9, 440.9 or ICD-10 I47.2, I49.0, I46, I50, I51.4, I51.5, I51.9, and I70.9).[33] Misclassification of CHD deaths into ill-defined cardiovascular disease codes occurs in many nations, resulting in gross underestimation of CHD mortality. Lozano et al. developed regression equations using standard CHD death rates and the relative risk of CHD attributable to national smoking prevalence to develop correction factors for the ill-defined cardiovascular code use in high- and low-miscoding nations.[33] Because the CHEFS codes less than 1% of all deaths into these ill-defined codes (well below the international median of 5%),[34] in our base model we employed a highly specific approach and attributed the small age- and sex- specific proportions of deaths categorized with ill-defined codes recommended by the World Health Organization for "low ill-defined cardiovascular disease coding" countries.[33,35] In a sensitivity analysis, we repeated our model simulation after calibrating the model to match the higher number of CHD deaths resulting when a larger proportion of ill-defined coded deaths are classified as CHD deaths (using correction factors advised for "high ill-defined cardiovascular disease coding" countries, Table 2) [33,35]

**Table 2:** Variability in the estimated proportions of deaths due to coronary heart disease in China.

**Age and sex category**	CHEFS* with conservative ICD definition of CHD (main assumption)†	**CHEFS with liberal ICD definition of CHD‡**	**Global Burden of Disease estimates, 2002**[36]
Men			
35–44 years	0.04	0.04	0.03
45–54	0.04	0.05	0.06
55–64	0.08	0.10	0.07
65–74	0.08	0.09	0.09
75–84	0.06	0.08	0.06

Women			
35–44 years	0.03	0.03	0.03
45–54	0.06	0.05	0.06
55–64	0.07	0.09	0.08
65–74	0.08	0.09	0.10
75–84	0.05	0.06	0.11

* China National Hypertension Survey Epidemiology Follow-up Study

†CHD defined as myocardial infarction (ICD-9 410, 412 or ICD-10 I21, I22), angina and other CHD (ICD-9 411, 413 and 414, or IC-10 I20, I23–I25), and a fixed proportion of "ill-defined" cardiovascular disease coded events and deaths.[33] (ICD-9 codes 427.1, 427.4, 427.5, 428, 429.0, 429.1, 429.2, 429.9, 440.9 or ICD-10 I47.2, I49.0, I46, I50, I51.4, I51.5, I51.9, and I70.9). The conservative definition of CHD assumed that very few CHD deaths were misclassified into ill-defined cardiovascular codes, so few of those deaths were counted as CHD deaths.

‡The more liberal coding method used the same ICD codes described above, but assumed that a higher proportion of CHD deaths were mis-coded into the "ill-defined" cardiovascular codes, so more deaths assigned these ill-defined codes are counted as CHD deaths.[33]

We assumed the same number of total deaths in China for the year 2002 reported by the Global Burden of Disease Study, [36] an estimate that adjusted for underreporting of deaths in the 2000 Chinese Census.[35,37] In our main simulation, we assumed age- and sex-specific proportions of total deaths attributable to CHD estimated by the nationally-representative CHEFS. Deaths identified in CHEFS survey households were verified using hospital records (when available) and death certificates. Cause of death was assessed and at times revised after review by an assessment committee in each province, and a study-wide end-point committee at the Chinese Academy of Medical Sciences in Beijing conducted an additional independent review before determining the cause of each death.[8] Global Burden of Disease Study cause-specific mortality data for China in 2002 were estimated from two distinct Chinese government sources: the Ministry of Health-Vital Registration System, and the Disease Surveillance Points system.[35] In order to demonstrate the impact of uncertainty regarding mortality assumptions and ensure comparability with the Global Burden Study and other studies,[38] we conducted a sensitivity analysis assuming the age-specific CHD mortality rates employed by the Global Burden of Disease Study for 2002. (Data from personal communication, Colin D. Mathers, Ph.D., April, 2008) The proportion of total deaths assigned to CHD assumed by the Global Burden Study was markedly higher in women of all ages compared with CHEFS estimates (Table 2).

### CHD Incidence, Prevalence, and Case-fatality

Age- and sex- specific incidence of CHD in the population with no prior CHD was based on the incidence observed in the CMCS cohort study. Rates of repeat CHD events in the population diagnosed with CHD was estimated from data from Canada and the U.S. [39-42] Initially, prevalence of CHD in the model's base year was estimated from the prevalence of a self-reported history of myocardial infarction (answered yes to "has your doctor told you that you had a heart attack?") or definite or possible angina recorded using the Rose questionnaire[43] in the InterASIA Study.[28] Because self-reported angina prevalence in the base year of 2000 was thought to be the most unreliable epidemiologic parameter (due possibility to a high number of false-positive angina diagnoses made with the Rose questionnaire [34,44]) the prevalences of self-reported angina and total CHD were adjusted to fit with angina (cases presenting for medical attention and coded ICD-9 413) and overall CHD incidence observed in the CMCS. Case-fatality rates increased by 1% annually in the MONICA Beijing population during 1984–1993,[45] but have been declining since (personal communication, Dong Zhao, M.D., Ph.D., April, 2008). In light of these dynamic changes, we took a conservative approach and assumed no case-fatality trend, but did model the consequences of assuming higher and lower case-fatality rates in sensitivity analyses. Incidence and fatality data over the years 1993–2004 were estimated from pooled Beijing Sino-MONICA Study data, and the main age-specific CHD case-fatality rate assumptions were estimated from the overall rates. Twenty-eight day case-fatality rates for ages 25–74 years in the Beijing Sino-MONICA population were 53% for Chinese men and 66% for Chinese women. These rates were lower than rates assumed for China in 2000 by the Global Burden of Disease Study – 62% for men and 72% for women.[34] In that analysis Global Burden investigators began with Sino-MONICA (Beijing) data from 1984–1993 and extrapolated these rates to the year 1996. We performed two sensitivity analysis: one that used the higher case-fatality rates assumed for China in the Global Burden study for 2000, and another using the lower case-fatalities observed in the Beijing Sino-MONICA population since 1999 [average over 1999–2004 for 25–74 year old men (40%) and women (49%)].

### CHD prediction and model calibration

The CHD Policy Model-China risk equation uses means and β coefficients for age, sex, systolic BP, smoking status, LDL, HDL, diabetes, and BMI. A separate non-CHD death equation includes age, sex, systolic BP, diabetes, and smoking status. Multivariate hazard ratios were estimated for CHD risk factors with age- and sex-specific Cox proportional hazards models using data from the CMCS, centered on age 50 years, with CHD or non-CHD death as the outcome.[27] (Tables 3 and 4) Measurement of CHD risk factors and formulation of Cox proportional hazards models in the CMCS is described in detail elsewhere.[27] In the Cox models, systolic BP, LDL cholesterol, HDL cholesterol, and BMI were treated as continuous variables. Statistically significant interactions of age with systolic BP and of age with smoking were incorporated into the final prediction model. The estimated risk for CHD from active smoking in Chinese women in the CMCS is thought to be unreliable (perhaps due the small number of female smokers in the cohort),[27] so the estimate for CHD risk from smoking in Chinese women participating in the PRC-USA cohort study[46] was substituted into the CHD risk equation, after adjusting the estimate to reflect risk for total CHD (myocardial infarction, angina, or arrest) rather than hard CHD (myocardial infarction or arrest only).

**Table 3:** Multivariate Cox proportional hazard model hazard ratios for CHD risk in Chinese adults aged 35–74, the CMCS

	**Males**		**Females**	
**Variable**	**Hazard Ratio**	**95%CI**	**Hazard Ratio**	**95%CI**
Age (10 years)	1.93	(1.57, 2.39)	1.99	(1.42, 2.79)
HDL (10 mg/dl or 0.26 mmol/l)	0.98	(0.88, 1.09)	0.78	(0.64, 0.93)
LDL (20 mg/dL or 0.51 mmol/l)	1.17	(1.10, 1.27)	1.11	(0.98, 1.24)
Systolic BP (20 mm Hg)	1.35	(1.17, 1.52)	1.27	(1.04, 1.55)
Diabetes (yes = 1, no = 0)	1.35	(0.82, 2.21)	2.43	(0.89, 3.47)
Active smoking (yes = 1, no = 0)	2.00	(1.45, 2.75)	2.51*	Not available†

Excepting smoking in women, estimates are from a multivariate model containing all variables shown in the table.

*Hazard ratio for active smoking in women from the PRC-USA cohort (Wu et al.)[46] after adjusting the estimate to reflect total CHD (angina, myocardial infarction, and arrest) in place of hard CHD (myocardial infarction and arrest only). See text for explanation.

† 95% confidence interval for the original estimate was not published.

**Table 4:** Multivariate Cox proportional hazard model hazard ratios for non-CHD mortality, Chinese men aged 35–74, the CMCS, 1992–2002.

	**Males**		**Females**	
**Variable**	**Hazard Ratio**	**95%CI**	**Hazard Ratio**	**95%CI**
Age (10 years)	2.03	(1.78, 2.35)	1.72	(1.43, 2.06)
Systolic BP (20 mm Hg)	1.25	(1.15, 1.37)	1.32	(1.17, 1.46)
Diabetes (yes = 1, no = 0)	1.25	(0.90, 1.73)	1.22	(0.77, 1.93)
Active smoking (yes = 1, no = 0)	1.37	(1.12, 1.67)	2.02	(1.26, 3.26)

Estimates are from a multivariate model containing all variables shown in the table.

The model's incidence was calibrated to reproduce the total number of deaths reported by the Global Burden of Disease Study for the year 2002 [36] under fixed case-fatality assumptions. Beginning with rates of CHD and non-CHD deaths reported from the CHEFS[8], we inflated CHD and non-CHD death rates proportionally (by 30%) in the model's simulation of the year 2002 to approximate the Global Burden of Disease estimate for that year. We then ensured that the Model's age-specific CHD and non-CHD death rates were constant over the 30-year simulation.

### Disability-adjusted life years (DALYs) lost due to CHD

DALYs were used to estimate the future burden of fatal and nonfatal CHD in China. The DALY measure combines Years of Life Lost (YLL) to premature death and the number of Years of life Lost due to Disability (YLD) using a set of disease-specific weights that value the level of disability. DALY disability weights range from zero (no disability) to one (death or extreme disability). In the absence of China-specific disability weights, the DALY weights established by the Global Burden of Disease study have the advantage of being generated in a standardized method and have been shown to be valid across cultures.[47]

YLL due to premature CHD death were calculated using a Global Burden of Disease calculator,[48] with age-specific life expectancies based on internationally standard life tables (West Model Levels 25 and 26 for men and women, respectively).[35] YLD were calculated by entering annual predictions of non-fatal acute myocardial infarctions, incident angina, and estimated incident heart failure. Non-fatal acute myocardial infarctions were defined as 28-day survivors of MI, a condition assumed to be disabling for a mean of 0.58 years with a DALY weight of 0.44.[34] Age- and sex-specific disability weights and disease durations were those assumed for China in 2004 for the Global Burden of Disease Study (personal communication, Colin Mathers, Ph.D., August, 2008). [34] Angina pectoris was assumed to persist for an average of 8.3 years in men (aged 35–84 years) and 9.4 years in women (aged 35–84) with a DALY weight of 0.16.[34,49] It was assumed that 20% of incident non-fatal myocardial infarctions led to congestive heart failure,[34,50] which then lasts for an average of 2.6 years in men (aged 35–84 years) and 3.3 years in women (aged 35–84 years) with a DALY weight of 0.25.[34,49] It was assumed that there was no remission from either angina or heart failure.

Total annual DALYs and DALYs stratified by age < 65 or ≥65 years are reported. The Global Burden Study time-discounted YLL and YLD at 3% annually after the year of interest in primary analyses and used age weights, which give less weight to younger and older ages in calculating DALYs.[49] We decided *a priori *not to use discounting or age weights in our primary estimates, but we do report discounted (3%) and age-weighted DALY estimates from sensitivity analyses.

### Predicting the impact of population growth and aging on CHD in China from 2000–2029

We used the CHD Policy Model-China to predict the effect of an aging and growing population on the epidemic of CHD in China over 2000–2029. We entered the population of China aged 35–84 in 2000 and the number of Chinese becoming 35 years old annually from 2001–2029.[1] Risk factors other than age were held constant at levels measured in 2000–2001.[28,51-55] Case-fatality rates were also held constant over future years. Annual absolute numbers and rates of CHD events, CHD deaths, and non-CHD deaths were tabulated over the 30 years of the simulation. CHD predictions for individual years were used to calculate YLL, YLD, and DALYs attributable to CHD for the years 2000, 2010, 2020, and 2030. We subsequently analyzed the independent effect of an aging population on future CHD by stratifying annual predictions of deaths and DALY by age 35–64 and age ≥ 65 years.

## Results

### CHD events and deaths in China, 2000–2029

For 2002, the 7,126,000 total deaths estimated from the CHD Policy Model-China for Chinese adults aged 35–84 matched the 7,126,000 deaths reported by the Global Burden of Disease Study for that year.[36] The Model estimated there were 1,084,000 CHD events, 457,000 CHD deaths, 472,000 non-fatal myocardial infarctions, and 227,000 new cases of angina pectoris in the model's base year of 2000.

With BP, cholesterol, BMI, diabetes, and smoking held at their 2000 levels, and case-fatality rates likewise held constant, we predicted an estimated 3.4 million CHD events and 1.4 million CHD deaths will occur over 2010–2020 in excess of the absolute numbers for 2000–2009 (Table 5), and 7.8 million excess CHD events and 3.4 million CHD deaths over 2020–2029 compared with the absolute numbers predicted for 2000–2009. There will be an estimated 64% increase in the number of CHD events in Chinese adults 35–84 years old in the third decade 2020–2029 compared with 2000–2009 (61% increase in men; 69% increase in women). With CHD risk factors other than age held constant, the number of non-CHD deaths in Chinese adults (including stroke deaths) was predicted to increase by 59% in 2020–2029 compared with 2000–2009 (Table 5).

**Table 5:** Main simulation: predicted coronary heart disease (CHD) events and deaths and non-coronary deaths in Chinese adults 35–84 years old within three successive decades, 2000–2029, the CHD Policy Model-China.

**Decade**	**Person-Years, China**	**Coronary Heart** **Disease Events**	**Coronary Heart** **Disease Deaths**	**Non-CHD Deaths**
**2000–2009**	Total 5,728,376,000	12,259,000	5,228,000	71,000,000
	Men 2,911,466,000	7,758,000	3,156,000	41,675,000
	Women 2,816,911,000	4,502,000	2,073,000	29,324,000

**2010–2019**	Total 6,867,272,000	15,650,000	6,670,000	89,936,000
	Men 3,445,402,000	9,843,000	4,020,000	52,707,000
	Women 3,421,870,000	5,806,000	2,650,000	37,229,000

**2020–2029**	Total 7,692,894,000	20,098,000	8,580,000	114,718,000
	Men 3,817,832,000	12,467,000	5,094,000	66,301,000
	Women 3,875,062,000	7,631,000	3,486,000	48,416,000

**2000–2029**	Total 20,288,542,000	48,007,000	20,478,000	275,654,000
	Men 10,174,670,000	30,068,000	12,269,000	160,684,000
	Women 10,113,843,000	17,939,000	8,209,000	114,970,000

**Figure 2:**
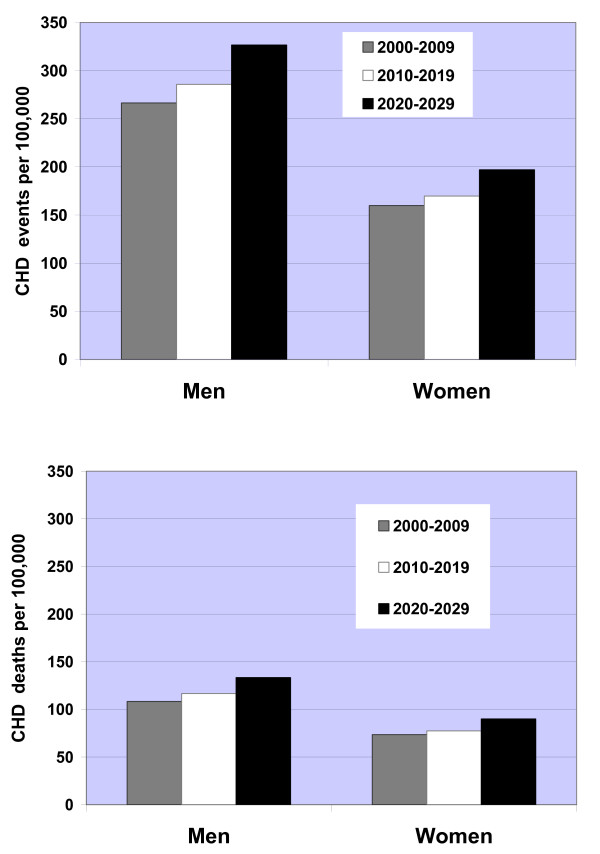
**a. Predicted ten year rates of coronary heart disease (CHD) events in Chinese men and women aged 35–84, 2000–2029 from the CHD Policy Model-China.** Rates are CHD events per 100,000 person-years. b. Predicted ten year rates of coronary heart disease (CHD) deaths in Chinese men and women aged 35–84, 2000–2029 from the CHD Policy Model-China. Rates are deaths per 100,000 person-years.

**Figure 3:**
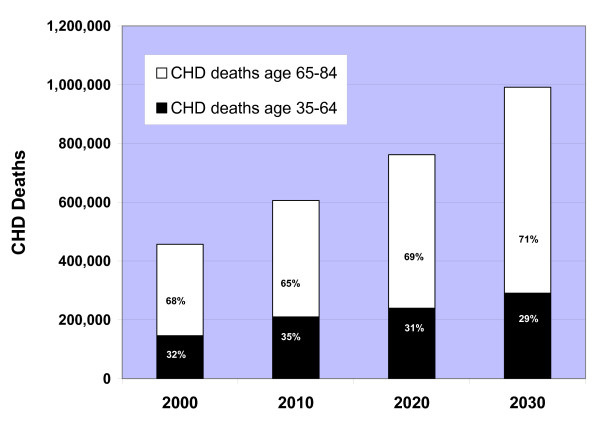
Predicted annual total CHD deaths by age <65 years or ≥ 65 years in Chinese adults 35–84 years old in 2000, 2010, 2020, and 2030, the CHD Policy Model-China.

The aging of the Chinese population was reflected in the predicted rise in the overall rates of CHD events and deaths, which were most pronounced in the third decade 2020–2029 (Figures 2a, b). Due to the aging of the Chinese population, CHD deaths in the simulation increased 68% in persons 65–84 years old in 2020–2029 compared with 2000–2009, whereas the increase in CHD deaths was only 57% in adults 35–64 years old. In 2000, 2010, 2020, and 2030, two-thirds or more of the growing number of annual CHD deaths occurred in persons ≥ 65 years old (Figure 3).

### Future burden of CHD death and disability in China

**Figure 4:**
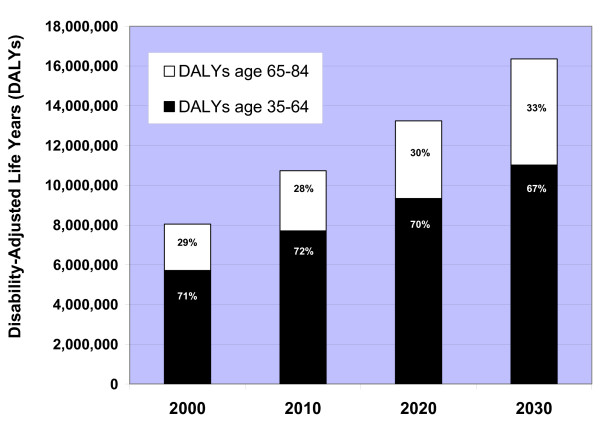
Predicted annual disability-adjusted life years (DALY) due to CHD by age <65 years or ≥ 65 years in Chinese adults 35–84 years old in 2000, 2010, 2020, and 2030, the CHD Policy Model-China.

Predicted annual DALYs attributable to CHD were largely driven by years of life lost due to CHD mortality, and annual DALYs increased with each successive decade in China (Table 6). When DALYs were stratified by age <65 or ≥ 65 years old, a greater number of DALYs and more than two-thirds of the burden of CHD was persistently concentrated in the population <65 years old, despite the higher proportions of CHD deaths in the population ≥ 65 (Figure 4).

**Table 6:** Main simulation: predicted years of life lost (YLL), years of life lost due to disability (YLD), and disability-adjusted life years (DALYs) attributable to CHD in Chinese adults aged 35–84 years, 2000, 2010, 2020, and 2030.

**Year**	Disability-Adjusted Life Years*	
**2000**	CHD deaths	457,000
	Incident non-fatal MI	472,000
	Incident angina	227,000
	Incident heart failure	94,000
	YLL	7,650,000
	YLD	392,000
	**DALYs**	**8,042,000**
	**DALYs per 1,000 persons**	**16.1**

**2010**	CHD deaths	606,000
	Incident non-fatal MI	618,000
	Incident angina	325,000
	Incident heart failure	124,000
	YLL	10,196,000
	YLD	534,000
	**DALY**	**10,730,000**
	**DALYs per 1,000 persons**	**16.5**

**2020**	CHD deaths	762,000
	Incident non-fatal MI	789,000
	Incident angina	366,000
	Incident heart failure	158,000
	YLL	12,639,000
	YLD	603,000
	**DALY**	**13,242,000**
	**DALYs per 1,000 persons**	**18.2**

**2030**	CHD deaths	992,000
	Incident non-fatal MI	1,015,000
	Incident angina	437,000
	Incident heart failure	203,000
	YLL	15,649,000
	YLD	706,000
	**DALY**	**16,356,000**
	**DALYs per 1,000 persons**	**20.4**

*DALY estimates reported in this table were not discounted and were not age weighted

### Sensitivity Analyses

When we assumed that a higher proportion of deaths assigned to ill-defined cardiovascular ICD codes were in fact due to CHD, we estimated 215,000 more CHD events and 76,000 more CHD deaths in the year 2000 than we did with our base model. Assuming the higher proportions of total deaths attributable to CHD reported by the Global Burden of Disease Study resulted in an additional 233,000 predicted CHD events and 96,000 predicted CHD deaths for 2000. Most of the excess was in women, in whom almost 50% more CHD deaths were predicted under this assumption compared with the main simulation. The pattern of additional CHD events and deaths under the more liberal ICD coding or the Global Burden Study's higher proportion of CHD deaths continued through the 30-year simulation, leading to a substantial cumulative number of incremental CHD events and deaths compared with the main simulation (Table 7). In the sensitivity analysis assuming higher CHD case-fatality rates, estimates of CHD events were identical to the base model in 2000, but there were 143,000 additional CHD deaths. Over a 30-year simulation, assuming higher case-fatality rates resulted in >20% higher cumulative mortality estimates (Table 7). Compared with the base model, overall CHD event rates diminished over time under a higher CHD case-fatality assumption, because while rates of new CHD events were unchanged, fewer chronic CHD patients in the Disease History portion of the model led to fewer repeat CHD events. Assuming the lower case-fatality rates observed recently in the Beijing Sino-MONICA population led to a similar estimated incidence but 20% fewer estimated CHD deaths. Assuming a lower case-fatality rate also led to a 12% higher predicted prevalence of CHD over the entire simulation (11% higher in men; 13% higher in women).

**Table 7:** Sensitivity analyses

**Decades**	**Sensitivity Analysis**	**Coronary Heart** **Disease Events**		**Coronary Heart** **Disease Deaths**		**Non-coronary Deaths**	
		**Incremental difference**	**%Change**	**Incremental difference**	**%Change**	**Incremental difference**	**%Change**

**2000–2029**	More liberal ICD definition of CHD*						
	Total	9,973,000	20.8	3,561,000	17.4	-3,270,000	-1.2
	Men	7,452,000	24.8	2,616,000	21.3	-2,605,000	-1.6
	Women	2,521,000	14.1	945,000	11.5	-665,000	-0.6

**2000–2029**	**Higher proportion of deaths due to CHD, Global Burden of Disease Study, 2002**[36]						
	Total	10,788,000	22.5	4,732,000	23.1	-4,683,000	-1.7
	Men	2,267,000	7.5	657,000	5.4	-723,000	-0.4
	Women	8,521,000	47.5	4,074,000	49.6	-3,960,000	-3.4

**2000–2029**	**Higher CHD case-fatality rate†**						
	Total	-3,031,243	-6.3	4,709,000	23.0	-1,075,000	-0.4
	Men	-1,787,000	-5.9	2,978,000	24.2	-762,000	-0.5
	Women	-1,244,000	-6.9	1,730,000	21.1	-313,000	-0.3

**2000–2029**	**Lower CHD case-fatality rate‡**						
	Total	324,000	1.5	-3,992,000	-19.5	814,000	<0.01
	Men	447,000	0.7	-2,289,000	-18.7	515,000	<0.01
	Women	-123,000	-0.7	-1,704,000	-20.8	299,000	<0.01

Incremental differences in predicted CHD events and non-CHD deaths compared with the main simulation in Chinese adults 35–84 years old over the years 2000–2029, under alternate mortality assumptions

* CHD defined as myocardial infarction (ICD-9 410, 412 or ICD-10 I21, I22), angina and other CHD (ICD-9 411, 413 and 414, or IC-10 I20, I23–I25), and a fixed proportion of "ill-defined" cardiovascular disease coded events and deaths.[33] (ICD-9 codes 427.1, 427.4, 427.5, 428, 429.0, 429.1, 429.2, 429.9, 440.9 or ICD-10 I47.2, I49.0, I46, I50, I51.4, I51.5, I51.9, and I70.9). The liberal coding method used the same ICD codes described above, but assumed that a higher proportion of CHD deaths were mis-coded into the "ill-defined" cardiovascular codes, so more deaths assigned these ill-defined codes are counted as CHD deaths.[33]

†Higher case-fatality rates assumed for China in 2000 by the Global Burden of Disease Study – age trended, overall 62% for men and 72% for women.[34]

‡Lower case-fatality rates observed in the Sino-MONICA Beijing population over 1999–2004 averaged for age 25–74 years – age trended, overall 40% for men and 49% for women (Personal communication, Dong Zhao, M.D., Ph.D., April, 2008)

Adding Global Burden of Disease age weights (but no discounting) led to lower estimates of 5.4, 7.2, 8.8, and 10.6 million CHD-related DALYs for the years 2000, 2010, 2020, and 2030, respectively. With age weights added, the projected proportions of total CHD DALYs attributable to the adult population <65 years old were 77% for 2000, 78% for 2010, 76% for 2020, and 73% for 2030. Discounting future DALYs at 3%, but using no age weights resulted in estimates of 6.1, 8.1, 10.1, and 12.6 million CHD DALYs for 2000, 2010, 2020, and 2030, respectively, and lower percentages of total CHD DALYs for the adult population <65 years old (67%, 68%, 67%, and 63%). Applying both a 3% discount to DALYs for future years and age weights led to the lowest CHD DALY estimates: 4.1, 5.5, 6.7, and 8.2 million for 2000, 2010, 2020, and 2030, respectively, and 74%, 75%, 73%, and 71% of total CHD DALYs attributed to the adult population <65 years old.

## Discussion

It is well known that the population of China is growing and aging. Using the CHD Policy Model-China, which incorporated China-specific data, we projected an excess of at least 7.8 million CHD events and 3.4 million CHD deaths over 2020–2029 compared with 2000–2009, even when holding levels of CHD risk factors constant, based on forecasted population growth and aging of the Chinese population.

The independent effect of aging on increasing CHD deaths will be greatest after 2020. Despite the more pronounced increase in CHD deaths in the population ≥ 65 years old, our DALY estimates indicated that the greatest direct disease burden of CHD will remain in the population <65. The impact of non-fatal CHD in older Chinese people will have a particularly profound indirect effect on their working-age children: the population of China is overall aging, approximately 70% of older parents are financially dependent on their children in China,[56] and current population policies will lead to a diminishing younger population able to support aging parents.[57] As a result, the aged dependency ratio (persons >65 years old/persons 15–64 years old) is expected to more than double in China between 2000 and 2029,[1] while increasing numbers of persons >65 years old will be living with CHD and other chronic diseases. In terms of the burden of CHD in working persons and the increasing dependency ratio, our results replicate those of Leeder et al.'s analysis of the macroeconomic impact of cardiovascular disease in China in the years 2000, 2030, and 2040.[9]

Ongoing cohort studies in China have demonstrated worsening trends in CHD risk factors, such as dyslipidemia, hypertension, obesity, and diabetes.[26,46,53,55,58-61] Smoking prevalence in Chinese men remains high at approximately 60%.[52] Although risk factor levels may continue to increase in a rapidly developing and urbanizing China, we held risk factors at their 2000 levels for the present analysis. We therefore may have underestimated the potential magnitude of the epidemic of CHD in China. On the other hand, if the decline in CHD case-fatality rates observed in the Beijing Sino-MONICA Study population from 1983–2004 is generalizeable to all of China, this might lead to overestimation of future CHD mortality, depending on the magnitude of increase in CHD incidence and decline in CHD case-fatality rate. A future trend in declining CHD case-fatality was not modeled in the primary analysis, but a sensitivity analysis assuming the lower CHD case-fatality rates observed recently in Beijing 1999–2004 predicted almost four million fewer CHD deaths over 2000–2029, but a 12% increase in CHD prevalence. Declines in case-fatality rates may continue due to increased uptake of CHD treatments,[11,62] or to more widespread diagnosis of less severe CHD cases. Recent declines in CHD case-fatality in China suggest that the trend toward declining CHD mortality and increased CHD prevalence documented in the U.S.[16,63,64] and other developed nations in recent decades may eventually occur in China.

Our model's projections rely heavily on reliable CHD mortality estimates for China, so we conducted sensitivity analyses to demonstrate the variability in the model's predictions under different cause-specific mortality assumptions. Though estimated cumulative numbers of CHD events and deaths increased substantially when we defined more ill-defined cardiovascular coded deaths as CHD or assumed the higher proportion of CHD deaths reported by the Global Burden of Disease Study, the same proportional increase in CHD outcomes over time persisted under these alternate mortality assumptions. The proportion of total deaths coded into ill-defined cardiovascular codes was less than 1% in the CHEFS, so misclassification due to improper ICD coding of CHD deaths did not likely bias our main simulation results. Our CHD mortality estimate for Chinese adults aged 35–84 years in 2002 (486,00 deaths) was more than 20% lower than the number reported by the Global Burden of Disease investigators for the same year and age range (590,000).[36] Overall mortality and population assumptions were the same, therefore differences between the Global Burden estimates and our main simulation results stem from their assumed higher proportion of CHD deaths, particularly in Chinese women. Official Chinese vital statistics are incomplete,[65,66] particularly in rural areas.[66] Global Burden of Disease cause of death estimates draw from the Chinese Ministry of Health-Vital Registration System and the Disease Surveillance Points system,[49] and the proportion of deaths attributable to CHD among the elderly differs by almost 3% between these two sources.[66] The CHEFS serves as the main source for our CHD mortality rate estimates. The CHEFS employed the same standard two-tiered mortality assessment protocol nationwide, and therefore may represent a more reliable source of age- and sex-specific cause of death estimates. Proposed improvements to death registration capacity, both globally[67] and in China,[66] may minimize future uncertainty regarding CHD mortality. Regarding our disability estimates, because older age is the most potent risk factor for CHD, our DALY estimates were dramatically lower when age weights were applied, though the trend toward increasing DALYs with time remained.

The main strength of the current analysis is that it modeled CHD in the entire adult population of China, resulting in a detailed examination of the impact of the growing and aging of the Chinese population on the future epidemic of CHD. However, our cardiovascular disease prediction model for China has limitations. Stroke currently is a far more common cause of death in China than is CHD. It is a major limitation that the current version of the CHD Policy Model-China includes the impact of stroke as a component of non-CHD mortality but does not specifically compute the annual or cumulative number of strokes. CHD prediction in the Policy Model-China relies heavily on a single Chinese cohort study. However, the Cox model coefficients from the CMCS are similar to those published from another Chinese cohort.[46] The Policy Model-China risk function was calibrated using nationally-representative Chinese mortality data, [8] and in this analysis, age- and sex-specific rates of CHD, CHD deaths, and non-CHD deaths remained stable over the 30 year simulation. Nonetheless, our model may not be fully representative of China, which is a large, multi-ethnic, and multi-cultural nation undergoing rapid change. Although the BRIG study included CHD patients from all over China in tertiary- and secondary-level hospitals, patient or hospital selection bias may have resulted in revascularization rates higher than those representative of China as a whole. The Rose questionnaire has not been validated as a tool for diagnosing angina in Chinese subjects, but our prevalence estimates relied less on Rose questionnaire-reported angina and more on incident angina identified in clinical settings (presenting for medical attention and coded as ICD-9 413 in the CMCS cohort). As a result, we may have excluded milder angina and underestimated the burden of angina in Chinese adults, especially in females.[68,69] Lastly, our estimates of CHD incidence may be biased by the fact that estimates of CHD events in the population diagnosed with CHD were derived from hospital databases and clinical trials describing North American populations and cohorts.

## Conclusion

Our results demonstrate the joint effects of population growth and aging on the CHD epidemic in China. The patterns of CHD events, CHD deaths, and disability-adjusted life years that we found suggest that while an aging Chinese population will lead to steep increases in the number of CHD events and deaths in persons ≥ 65 years old in the coming decades of this century, the predominant burden of CHD will continue to rest on the working population <65 year old.

## Abbreviations

CHD: coronary heart disease; DALY: disability-adjusted life year; BMI: body mass index; LDL: low density lipoprotein cholesterol; HDL: high density lipoprotein cholesterol; BP: blood pressure; WHO: World Health Organization; MONICA: Monitoring Trends and Determinants in Cardiovascular Disease project; CMCS: Chinese Multi-provincial Cohort Study; InterASIA: International Collaborative Study of Cardiovascular Disease in Asia Study; CHEFS: China National Hypertension Survey Epidemiology Follow-up Study; BRIG: Bridging the Gap in CHD secondary prevention study; PCI: percutaneous coronary intervention; CABG: coronary artery bypass graft surgery.

## Competing interests

The authors declare that they have no competing interests.

## Authors' contributions

AM conceived of the study, selected the computer model inputs, adapted and calibrated the computer model, conducted the analysis, and authored the first draft of the manuscript. DZ participated in the study design, supervised data analyses and contributed editorially to the manuscript, DG participated in the study design and contributed editorially to the manuscript, PC participated in the study design, consulted on the adaptation, calibration, and analytic use of the computer model and contributed editorially to the manuscript, C C-S conducted statistical analyses, JC participated in the study design, conducted statistical analyses and contributed editorially to the manuscript, JL contributed editorially to the manuscript, JH participated in the study design, supervised data analyses, and contributed editorially to the manuscript, LG assisted with conception of the study, participated in model and study design, and had final editorial approval for the finished manuscript.

## Pre-publication history

The pre-publication history for this paper can be accessed here:



## Supplementary Material

Additional file 1 **Supplementary appendix**. Additional materials describing the model assumptions and methods. Click here for file

## References

[B1] International Database, U.S. Census Bureau. http://www.census.gov/ipc/www/idb/.

[B2] National Bureau of Statistics of China. http://www.stats.gov.cn/english/.

[B3] Wang L, Kong L, Wu F, Bai Y, Burton R (2005). Preventing chronic diseases in China. Lancet.

[B4] Omran AR (1971). The epidemiologic transition. A theory of the epidemiology of population change. Milbank Mem Fund Q.

[B5] Yusuf S, Reddy S, Ounpuu S, Anand S (2001). Global burden of cardiovascular diseases: part I: general considerations, the epidemiologic transition, risk factors, and impact of urbanization. Circulation.

[B6] Murray CJ, Lopez AD (1997). Alternative projections of mortality and disability by cause 1990–2020: Global Burden of Disease Study. Lancet.

[B7] Gaziano TA (2007). Reducing the growing burden of cardiovascular disease in the developing world. Health Aff (Millwood).

[B8] He J, Gu D, Wu X, Reynolds K, Duan X, Yao C, Wang J, Chen CS, Chen J, Wildman RP, Klag MJ, Whelton PK (2005). Major causes of death among men and women in China. New Engl J Med.

[B9] Leeder S, Raymond S, Greenberg H, Liu H, Esson K (2004). A Race Against Time: the Challenge of Cardiovascular Disease in Developing Countries.

[B10] Murray CJ, Lopez AD (1997). Mortality by cause for eight regions of the world: Global Burden of Disease Study. Lancet.

[B11] Critchley J, Liu J, Zhao D, Wei W, Capewell S (2004). Explaining the increase in coronary heart disease mortality in Beijing between 1984 and 1999. Circulation.

[B12] Weinstein MC, Coxson PG, Williams LW, Pass TM, Stason WB, Goldman L (1987). Forecasting coronary heart disease incidence, mortality, and cost: the Coronary Heart Disease Policy Model. Am J Public Health.

[B13] Tosteson AN, Weinstein MC, Hunink MG, Mittleman MA, Williams LW, Goldman PA, Goldman L (1997). Cost-effectiveness of populationwide educational approaches to reduce serum cholesterol levels. Circulation.

[B14] Tosteson AN, Weinstein MC, Williams LW, Goldman L (1990). Long-term impact of smoking cessation on the incidence of coronary heart disease. Am J Public Health.

[B15] Prosser LA, Stinnett AA, Goldman PA, Williams LW, Hunink MG, Goldman L, Weinstein MC (2000). Cost-effectiveness of cholesterol-lowering therapies according to selected patient characteristics. Ann Intern Med.

[B16] Hunink MG, Goldman L, Tosteson AN, Mittleman MA, Goldman PA, Williams LW, Tsevat J, Weinstein MC (1997). The recent decline in mortality from coronary heart disease, 1980–1990. The effect of secular trends in risk factors and treatment. JAMA.

[B17] Goldman L, Coxson P, Hunink MG, Goldman PA, Tosteson AN, Mittleman M, Williams L, Weinstein MC (1999). The relative influence of secondary versus primary prevention using the National Cholesterol Education Program Adult Treatment Panel II guidelines. J Am Coll Cardiol.

[B18] Goldman L, Weinstein MC, Goldman PA, Williams LW (1991). Cost-effectiveness of HMG-CoA reductase inhibition for primary and secondary prevention of coronary heart disease. JAMA.

[B19] Goldman L, Weinstein MC, Williams LW (1989). Relative impact of targeted versus populationwide cholesterol interventions on the incidence of coronary heart disease. Projections of the Coronary Heart Disease Policy Model. Circulation.

[B20] Gaspoz JM, Coxson PG, Goldman PA, Williams LW, Kuntz KM, Hunink MG, Goldman L (2002). Cost effectiveness of aspirin, clopidogrel, or both for secondary prevention of coronary heart disease. New Engl J Med.

[B21] Edelson JT, Weinstein MC, Tosteson AN, Williams L, Lee TH, Goldman L (1990). Long-term cost-effectiveness of various initial monotherapies for mild to moderate hypertension. JAMA.

[B22] Bibbins-Domingo K, Coxson P, Pletcher MJ, Lightwood J, Goldman L (2007). Adolescent overweight and future adult coronary heart disease. New Engl J Med.

[B23] Bibbins-Domingo K, Coxson P, Pletcher MJ, Lightwood J, Goldman L, Appendix to (2007). Adolescent overweight and future adult coronary heart disease. N Engl J Med.

[B24] Wu Z, Yao C, Zhao D, Wu G, Wang W, Liu J, Zeng Z, Wu Y (2001). Sino-MONICA project: a collaborative study on trends and determinants in cardiovascular diseases in China, Part I: morbidity and mortality monitoring. Circulation.

[B25] Wu Z, Yao C, Zhao D, Wu G, Wang W, Liu J, Zeng Z (2004). Cardiovascular disease risk factor levels and their relations to CVD rates in China – results of Sino-MONICA project. Eur J Cardiovasc Prev Rehabil.

[B26] Liu SZD, Wang W, Liu J, Qin L, Zeng Z, Wu Z (2006). The trends of cardiovascular risk factors in urban and rural areas of Beijing during 1984–1999. Journal of Cardiovascular and Pulmonary Diseases.

[B27] Liu J, Hong Y, D'Agostino RB, Wu Z, Wang W, Sun J, Wilson PW, Kannel WB, Zhao D (2004). Predictive value for the Chinese population of the Framingham CHD risk assessment tool compared with the Chinese Multi-Provincial Cohort Study. JAMA.

[B28] He J, Neal B, Gu D, Suriyawongpaisal P, Xin X, Reynolds R, MacMahon S, Whelton PK (2004). International collaborative study of cardiovascular disease in Asia: design, rationale, and preliminary results. Ethn Dis.

[B29] Framingham Heart Study CD-ROM: Department of Health and Human Services; 2005.

[B30] Roger VL, Jacobsen SJ, Weston SA, Goraya TY, Killian J, Reeder GS, Kottke TE, Yawn BP, Frye RL (2002). Trends in the incidence and survival of patients with hospitalized myocardial infarction, Olmsted County, Minnesota, 1979 to 1994. Ann Intern Med.

[B31] Chambless L, Keil U, Dobson A, Mahonen M, Kuulasmaa K, Rajakangas AM, Lowel H, Tunstall-Pedoe H (1997). Population versus clinical view of case fatality from acute coronary heart disease: results from the WHO MONICA Project 1985–1990. Multinational Monitoring of Trends and Determinants in Cardiovascular Disease. Circulation.

[B32] Goodkind DM (2004). China's missing children: the 2000 census underreporting surprise. Population Studies.

[B33] Lozano R, Murray CJL, Lopez AD, Satoh T (2001). Miscoding and misclassification of ischaemic heart disease mortality. Global Programme on Evidence for Health Policy Working Paper No. 12. Geneva, World Health Organization.

[B34] Mathers CD, Truelson T, Begg S, Satoh T (2004). Global burden of ischemic heart diseae in the year 2000. Global Burden of Disease 2000 Working Paper. Geneva, World Health Organization.

[B35] Mathers C, Lopez AD, Murray CJL (2006). The Burden of Disease and Mortality by Condition: Data, Methods, and Results for 2001. Global Burden of Disease and Risk Factors.

[B36] Global Burden of Disease Study. Death and DALY estimates for 2002 by cause for WHO Member States. http://www.who.int/evidence/bod/en/.

[B37] Banister J, Hill K (2004). Mortality in China 1964–2000. Population studies.

[B38] Liu BQ, Peto R, Chen ZM, Boreham J, Wu YP, Li JY, Campbell TC, Chen JS (1998). Emerging tobacco hazards in China: 1. Retrospective proportional mortality study of one million deaths. BMJ (Clinical research ed).

[B39] Cannon CP, Battler A, Brindis RG, Cox JL, Ellis SG, Every NR, Flaherty JT, Harrington RA, Krumholz HM, Simoons ML, Werf FJ Van De, Weintraub WS, Mitchell KR, Morrisson SL, Brindis RG, Anderson HV, Cannom DS, Chitwood WR, Cigarroa JE, Collins-Nakai RL, Ellis SG, Gibbons RJ, Grover FL, Heidenreich PA, Khandheria BK, Knoebel SB, Krumholz HL, Malenka DJ, Mark DB, McKay CR, Passamani ER, Radford MJ, Riner RN, Schwartz JB, Shaw RE, Shemin RJ, van Fossen DB, Verrier ED, Watkins MW, Phoubandith DR, Furnelli T (2001). American College of Cardiology key data elements and definitions for measuring the clinical management and outcomes of patients with acute coronary syndromes. A report of the American College of Cardiology Task Force on Clinical Data Standards (Acute Coronary Syndromes Writing Committee). J Am Coll Cardiol.

[B40] Canto JG, Rogers WJ, Chandra NC, French WJ, Barron HV, Frederick PD, Maynard C, Every NR (2002). The association of sex and payer status on management and subsequent survival in acute myocardial infarction. Arch Intern Med.

[B41] Fu Y, Chang WC, Mark D, Califf RM, Mackenzie B, Granger CB, Topol EJ, Hlatky M, Armstrong PW (2000). Canadian-American differences in the management of acute coronary syndromes in the GUSTO IIb trial: one-year follow-up of patients without ST-segment elevation. Global Use of Strategies to Open Occluded Coronary Arteries (GUSTO) II Investigators. Circulation.

[B42] Every NR, Frederick PD, Robinson M, Sugarman J, Bowlby L, Barron HV (1999). A comparison of the national registry of myocardial infarction 2 with the cooperative cardiovascular project. J Am Coll Cardiol.

[B43] Rose GABH, Gillum RF, Princeas RJ (1982). Cardiovascular Survey Methods.

[B44] Garber CE, Carleton RA, Heller GV (1992). Comparison of "Rose Questionnaire Angina" to exercise thallium scintigraphy: different findings in males and females. J Clin Epidemiol.

[B45] Tunstall-Pedoe H, Kuulasmaa K, Mahonen M, Tolonen H, Ruokokoski E, Amouyel P (1999). Contribution of trends in survival and coronary-event rates to changes in coronary heart disease mortality: 10-year results from 37 WHO MONICA project populations. Monitoring trends and determinants in cardiovascular disease. Lancet.

[B46] Wu Y, Liu X, Li X, Li Y, Zhao L, Chen Z, Li Y, Rao X, Zhou B, Detrano R, Liu K (2006). Estimation of 10-year risk of fatal and nonfatal ischemic cardiovascular diseases in Chinese adults. Circulation.

[B47] Salomon J, Mathers CD, Chatterji S, Sadana R, Ustun TB, Murray CJL, Murray C, Evans D (2003). Quantifying Individual Levels of Health: Definitions, Concepts, and Measurement Issues. Health Systems Performance Assessment: Debate, Methods, and Empiricism.

[B48] Resources for National Burden of Disease Studies. http://www.who.int/healthinfo/global_burden_disease/en/index.html.

[B49] Mathers C, Lopez AD, Murray CJL, Lopez AD, Mathers CD, Ezzati M, Jamison DT, Murray CJL (2006). The Burden of Disease and Mortality by Condition: Data, Methods, and Results for 2001. Global Burden of Disease and Risk Factors.

[B50] Cowie MR, Mosterd A, Wood DA, Deckers JW, Poole-Wilson PA, Sutton GC, Grobbee DE (1997). The epidemiology of heart failure. Eur Heart J.

[B51] Gu D, Gupta A, Muntner P, Hu S, Duan X, Chen J, Reynolds RF, Whelton PK, He J (2005). Prevalence of cardiovascular disease risk factor clustering among the adult population of China: results from the International Collaborative Study of Cardiovascular Disease in Asia (InterAsia). Circulation.

[B52] Gu D, Wu X, Reynolds K, Duan X, Xin X, Reynolds RF, Whelton PK, He J (2004). Cigarette smoking and exposure to environmental tobacco smoke in China: the international collaborative study of cardiovascular disease in Asia. Am J Public Health.

[B53] He J, Gu D, Reynolds K, Wu X, Muntner P, Zhao J, Chen J, Liu D, Mo J, Whelton PK (2004). Serum total and lipoprotein cholesterol levels and awareness, treatment, and control of hypercholesterolemia in China. Circulation.

[B54] Gu D, Reynolds K, Wu X, Chen J, Duan X, Muntner P, Huang G, Reynolds RF, Su S, Whelton PK, He J (2002). Prevalence, awareness, treatment, and control of hypertension in china. Hypertension.

[B55] Gu D, Reynolds K, Duan X, Xin X, Chen J, Wu X, Mo J, Whelton PK, He J (2003). Prevalence of diabetes and impaired fasting glucose in the Chinese adult population: International Collaborative Study of Cardiovascular Disease in Asia (InterASIA). Diabetologia.

[B56] Sun F (1998). Ageing of the population in China: trends and implications. Asia-Pacific population journal/United Nations.

[B57] Hesketh T, Lu L, Xing ZW (2005). The effect of China's one-child family policy after 25 years. New Engl J Med.

[B58] Wu X, Duan X, Gu D, Hao J, Tao S, Fan D (1995). Prevalence of hypertension and its trends in Chinese populations. Int J Cardiol.

[B59] Gu D, Reynolds K, Wu X, Chen J, Duan X, Reynolds RF, Whelton PK, He J (2005). Prevalence of the metabolic syndrome and overweight among adults in China. Lancet.

[B60] Wang H, Du S, Zhai F, Popkin BM (2007). Trends in the distribution of body mass index among Chinese adults, aged 20–45 years (1989–2000). Int J Obesity (2005).

[B61] Wildman RP, Gu D, Muntner P, Wu X, Reynolds K, Duan X, Chen CS, Huang G, Bazzano LA, He J (2008). Trends in Overweight and Obesity in Chinese Adults: Between 1991 and 1999–2000. Obesity (Silver Spring).

[B62] Tunstall-Pedoe H, Vanuzzo D, Hobbs M, Mahonen M, Cepaitis Z, Kuulasmaa K, Keil U (2000). Estimation of contribution of changes in coronary care to improving survival, event rates, and coronary heart disease mortality across the WHO MONICA Project populations. Lancet.

[B63] Ergin A, Muntner P, Sherwin R, He J (2004). Secular trends in cardiovascular disease mortality, incidence, and case fatality rates in adults in the United States. Am J Med.

[B64] Rosamond WD, Chambless LE, Folsom AR, Cooper LS, Conwill DE, Clegg L, Wang CH, Heiss G (1998). Trends in the incidence of myocardial infarction and in mortality due to coronary heart disease, 1987 to 1994. New Engl J Med.

[B65] Mahapatra P, Shibuya K, Lopez AD, Coullare F, Notzon FC, Rao C, Szreter S (2007). Civil registration systems and vital statistics: successes and missed opportunities. Lancet.

[B66] Rao C, Lopez AD, Yang G, Begg S, Ma J (2005). Evaluating national cause-of-death statistics: principles and application to the case of China. Bulletin of the World Health Organization.

[B67] Setel PW, Macfarlane SB, Szreter S, Mikkelsen L, Jha P, Stout S, AbouZahr C (2007). A scandal of invisibility: making everyone count by counting everyone. Lancet.

[B68] Hemingway H, Langenberg C, Damant J, Frost C, Pyorala K, Barrett-Connor E (2008). Prevalence of angina in women versus men: a systematic review and meta-analysis of international variations across 31 countries. Circulation.

[B69] Timmis AD, Feder G, Hemingway H (2007). Prognosis of stable angina pectoris: why we need larger population studies with higher endpoint resolution. Heart (British Cardiac Society).

