# Supplementary material for: The future impact of population growth and aging on coronary heart disease in China: projections from the Coronary Heart Disease Policy Model-China
Source: BMC Public Health. 2008 Nov 27;8:394. doi: 10.1186/1471-2458-8-394 (PMC2631484; doi:10.1186/1471-2458-8-394)
Supplement: Additional file 1 — Supplementary appendix. Additional materials describing the model assumptions and methods. [file 1471-2458-8-394-S1.pdf]

Supplementary Appendix

This appendix has been provided by the authors to give readers additional information about their work.

Supplement to: Moran A, Zhao D, Gu D, et al. The Future Impact of Population Growth and Aging on Coronary Heart Disease in China: Projections from the Coronary Heart Disease Policy Model-China.

A1. Population Assumptions

The US Census Bureau international database[1] was used as the source for estimates of the adult Chinese population age 35-84 years (Appendix Table 1 below) because 1) the population estimates by age/sex for adults aged 35-84 in 2000 were quite similar to the estimates from the Chinese Census of 2000,[2] and projections of future 35 year olds (Table 2 below) were available from the US Census database and not the Chinese National Bureau of Statistics.

Appendix Table 1. Population of China aged 35-84 years in the year 2000, by age and sex, U.S. Census Bureau International Database (used in the CHD Policy Model-China).

Population by Age and Sex, 2000

	Midyear population		
Age	Both sexes	Male	Female
35-39	102,588,845	52,588,414	50,000,431
40-44	82,102,517	42,636,756	39,465,761
45-49	83,933,762	43,095,380	40,838,382
50-54	61,044,234	31,699,045	29,345,189
55-59	45,869,786	23,875,932	21,993,854
60-64	40,827,108	21,152,731	19,674,377
65-69	34,703,549	17,560,938	17,142,611
70-74	24,986,765	12,141,707	12,845,058
75-79	15,806,768	7,084,405	8,722,363
80-84	7,938,300	3,212,400	4,725,900
85-89	3,130,048	1,102,901	2,027,147
90-94	832,031	233,206	598,825

Appendix Table 2. Projected population of China aged 35 years in the years 2001-2029, by sex, U.S. Census Bureau International Database (used in the CHD Policy Model-China).

YEAR	Males	Females
2001	12,702,898	12,055,011
2002	11,611,867	10,932,090
2003	12,521,827	11,735,205
2004	13,764,163	13,065,102
2005	13,149,400	12,564,667
2006	13,236,969	12,368,116
2007	12,317,627	11,634,396
2008	12,073,845	11,646,067
2009	11,503,094	11,132,997
2010	10,587,460	10,217,905
2011	10,003,553	9,626,462
2012	9,461,310	9,090,618
2013	9,201,776	8,851,939
2014	9,437,871	9,046,089
2015	9,416,390	8,979,101
2016	9,086,373	8,576,822
2017	12,043,428	11,430,496
2018	10,044,172	9,746,499
2019	9,830,067	9,448,556
2020	9,867,333	9,354,966
2021	10,989,583	10,448,874
2022	13,056,164	12,418,353
2023	12,360,313	11,697,069
2024	12,598,862	11,808,453
2025	13,412,158	12,202,202
2026	11,011,614	10,219,689
2027	10,443,485	9,641,889
2028	10,168,950	9,338,966
2029	10,181,505	9,300,104

A2. Mortality rate assumptions for China in 2002.

The CHD Policy Model-China defined CHD as myocardial infarction (ICD-9 410, 412 or ICD-10 I21, I22), angina and other CHD (ICD-9 411, 413 and 414, or IC-10 I20, I23-I25), and a fixed proportion of “ill-defined” cardiovascular disease coded events and deaths (ICD-9 codes 427.1, 427.4, 427.5, 428, 429.0, 429.1, 429.2, 429.9, 440.9 or ICD-10 I47.2, I49.0, I46, I50, I51.4, I51.5, I51.9, and I70.9). The proportion of ill-defined cardiovascular disease coded deaths apportioned to CHD under liberal, “high ill-defined cardioscular disease

coding" and conservative, "low ill-defined coding" assumptions are listed below, according to the method developed by Lozano et al.[3] The more conservative definition was used re-apportion ill-defined coded cardiovascular disease deaths to CHD deaths in the main model.

Appendix Table 3. Proportion of deaths coded with ICD-9 codes 427.1, 427.4, 427.5, 428, 429.0, 429.1, 429.2, 429.9, 440.9 re-allocated toward CHD deaths (based on Lozano et al.)[3]

Males	Conservative ('low ill-defined coding); main assumption	Liberal ('high ill-defined coding)	Females	Conservative ('low ill-defined coding); main assumption	Liberal ('high ill-defined coding)
35-44	0.107	0.107	35-44	0	0
45-54	0.0395	0.4845	45-54	0.0505	0.2435
55-64	0.1545	0.8475	55-64	0.129	0.6745
65-74	0.2585	0.7795	65-74	0.2265	0.5420
75-84	0.1315	0.409	75-84	0.115	0.2780

Target total mortality for the Chinese adults aged 35-84 years in the year 2002 were taken from the World Health Organization (WHO) estimates: 3.9 million total deaths for men and 3.3 million for women. Age and sex-specific CHD and non-CHD mortality rates were estimated from the Chinese National Hypertension Epidemiology Follow-up Survey (CHEFS)[4] and then inflated by 30% to fit the 'envelope' of the WHO absolute mortality estimates.

Appendix Table 4. Original CHD deaths rates based on CHEFS estimates and inflated CHD death rates used in the CHD Policy Model-China. Rates are deaths/100,000 person-years.

Males	Original CHEFS CHD death rates	Inflated CHD death rates	Females	Original CHEFS CHD death rates	Inflated CHD death rates
35-44	9.2	12	35-44	3.8	5
45-54	19.8	26	45-54	13.0	17
55-64	108.1	141	55-64	57.3	74
65-74	275.1	358	65-74	181.3	236
75-84	522.3	679	75-84	345.3	449

A3. Incidence of CHD in persons with no prior diagnosis of CHD.

CHD incidence in men and women aged 35-84 years with no prior CHD diagnosis was based on 10-year incidence rates from the Chinese Multi-provincial Cohort Study (CMCS),[5] and calibrated to fit with CHD mortality and case-fatality assumptions.

Appendix Table 5. Calibrated incidence of CHD in persons free of CHD, the CHD Policy Model-China. Rates are cases of CHD/100,000 person-years.

**Males**	Policy Model-China CHD incidence	**Females**	Policy Model-China CHD incidence
35-44	50	35-44	20
45-54	75	45-54	40
55-64	350	55-64	170
65-74	670	65-74	420
75-84	700	75-84	550

A4. Prevalence of angina and total CHD

The Rose questionnaire has never been validated in a Chinese population. Results from one study performed in United Kingdom South Asians and Europeans suggest that the Rose questionnaire, or at least a diagnosis of 'definite angina' defined by the questionnaire may not be reproducible across populations.[6] We suspect that Rose questionnaire self-reported angina from the InterASIA study[7] likely included many false positives, a conclusion reached by others regarding Rose-diagnosed angina in their studies.[8, 9] For the CHD Policy Model-China, prevalence was calibrated to match incidence, case-fatality, and mortality assumptions. In particular, the prevalence of angina was driven predominantly by the incidence of angina (ICD-9 413) observed in the Chinese Multi-provincial Cohort Study (CMCS, see table below). In the CMCS, incident angina was defined based on cases of angina presenting for medical attention. The unreliable estimate for incidence in females 65-74 years old was fitted to known age trends from the Framingham Study.[10]

Appendix Table 6. Angina Incidence in the CMCS, China, 1990-2001.

Sex/Age Group	CMCS incidence of angina pectoris (ICD-9 413) in events/100,000 person-years
M 35-44	28.0
M 45-54	16.8
M 55-64	48.7
M 65-74	85.0
M 75-84	Not available
F 35-44	14.2
F 45-54	32.5
F 55-64	65.2
F 65-74	24.7 *
F 75-84	Not available

*Unreliable, based on few events

Given the overall lower CHD incidence in China, the Policy Model-China's age-specific prevalence of total CHD assumed for our model for 2000 correlates well with the % prevalence of angina pectoris assumed for the CHD Policy Model-U.S. for the U.S. in 2000 and that assumed for the 1996 U.S. Burden of Disease Study (see table below). The China Model also assumes that prevalence of angina correlates with overall CHD incidence, so that the prevalence of angina is lower in Chinese women than Chinese men. This is also the pattern assumed by the US Model and the US Burden of Disease Study.

Appendix Table 7. Comparison of angina prevalence assumed for the CHD Policy Model-China with Rose questionnaire-diagnosed angina (InterASIA Study 2000-2001) and angina prevalence assumed for the United States for the CHD Policy Model-USA and the U.S. Global Burden Study.

Sex/Age Group	InterASIA prevalence of angina (using the Rose questionnaire)	CHDPM-China 2000 angina prevalence assumption (fitted to incident angina in CMCS)	CHDPM-USA 2000 angina prevalence	GBD USA 1996 angina prevalence (approximations from Figure 4, Mathers et al.)[9]
M 35-44	2.4	0.03	1.06	0.1
M 45-54	2.7	0.54	3.24	1.2
M 55-64	4.5	0.85	5.87	3.2
M 65-74	3.0	2.67	8.71	5.0
M 75-84	Not available	4.38	11.26	7.0
F 35-44	4.2	0.03	0.74	0.2
F 45-54	6.0	0.43	1.73	0.3
F 55-64	4.5	0.51	3.44	1.0
F 65-74	5.7	1.51	6.20	3.5
F 75-84	Not available	3.10	9.26	6.2

A5. Case-fatality rate assumptions

Main CHD Policy Model-China 28-day case-fatality assumptions were estimated from pooled Beijing Sino-MONICA Study data from 1993-2004 (personal communication, Dong Zhao, MD, PhD, 2006) and the main age-specific CHD case-fatality rate assumptions were estimated from the overall rates. Twenty-eight day case-fatality rates for ages 25-74 years in the Beijing Sino-MONICA population were 53% for Chinese men and 66% for Chinese women. Age specific total CHD (including incident myocardial infarction (MI), cardiac arrest, and angina) and 'hard' CHD (MI and cardiac arrest only) 28-day case fatality rates assumed for the CHD Policy Model-China are listed below.

Appendix Table 8. Age-specific 28-day CHD mortality rates assumed for the CHD Policy Model-China

**Males**	Policy Model-China 28-day total CHD Case-fatality*	Policy Model-China 28-day hard CHD Case-fatality	**Females**	Policy Model-China 28-day total CHD Case-fatality	Policy Model-China 28-day hard CHD Case-fatality
35-44	0.18	0.38	35-44	0.28	0.61
45-54	0.28	0.46	45-54	0.32	0.53
55-64	0.37	0.48	55-64	0.42	0.60
65-74	0.46	0.55	65-74	0.54	0.63
75-84	0.60	0.67	75-84	0.73	0.76

*Total CHD includes angina, MI, and arrest. Hard CHD includes MI and arrest only.

Case-fatality rates increased by 1% annually in Chinese men and women in the MONICA Beijing population during 1984-1993,[11] but have been declining since (personal communication, Dr. Dong Zhao, 2006, see figure below). In light of these dynamic changes, we took the conservative approach of assuming no change in age-specific CHD case-fatality, but did model the consequences of assuming higher (similar to the 1984-1993 Beijing rates) and lower (similar to Beijing rates 1999-2004) case-fatality rates.

Appendix Figure 1. CHD Case-fatality, Beijing-MONICA 1984-2004 (unpublished data)

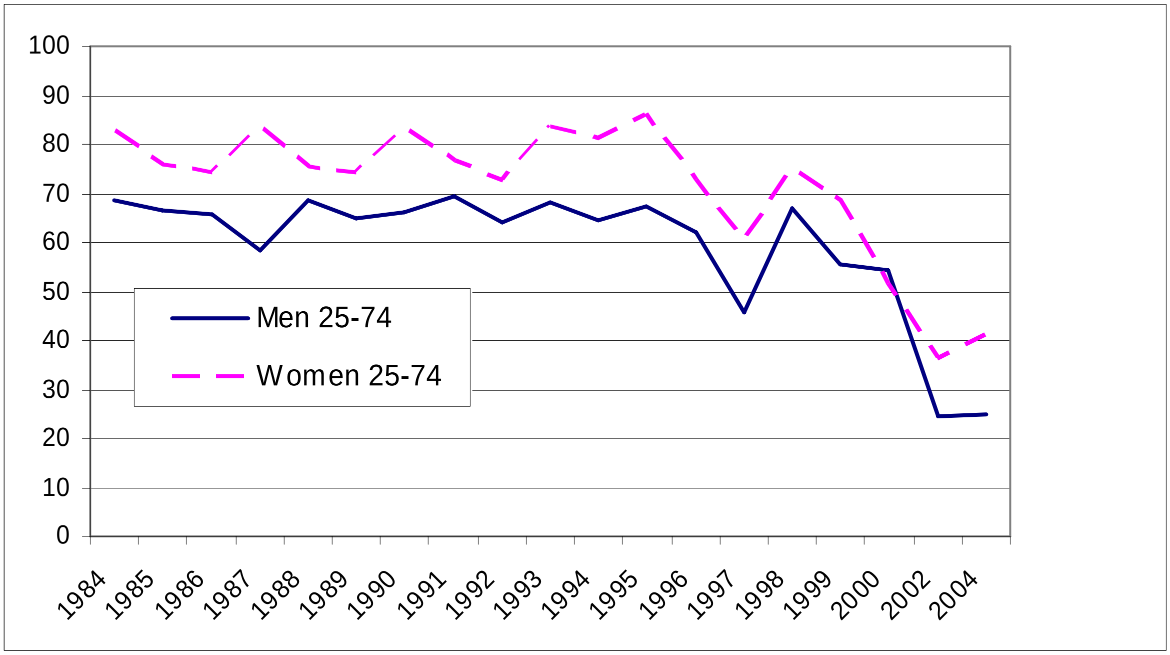

A6. Transition rates between risk factors

Transfers from one risk factor level to another were included to preserve the InterASIA Study proportions of the population with each risk factor level. For example, the proportion of 35-44 year old men with low (<100 mg/dL) LDL cholesterol is 0.482. For 45-54 year old men the proportion is 0.437. The shift toward higher LDL cholesterol levels is most likely caused by increasing LDL levels as people age. In higher age ranges, this trend reverses, so that by age 75-84, the proportion is 0.403. The change in the upper age ranges is most likely due to a more complex array of factors, including the fact that people with higher risk are more likely to die. Annual transfer rates between risk factor levels were calculated to reduce the low risk population from 0.482 to 0.437 over 10 years, without regard to the reason for the change, but taking into account the effect of the Model's CHD incidence and non-CHD death rates.

A7. Durations of non-fatal CHD sequelae: angina and congestive heart failure

For incident cases of angina and congestive heart failure, we assumed the age-specific mean durations assumed in 2004 by the WHO for the WPRO region, 90% of which comprises China (table below, personal communication, Colin Mathers, PhD, August, 2008).

Appendix Table 9. Durations of sequelae: angina pectoris (AP) and congestive heart failure (CHF) assumed for the WPR B1 region, the Global Burden of Disease Study, 2004.

WPR B1 2004		AP	CHF
age	sex	Duration (years)	Duration (years)
0	male	42.3827	0
5	male	33.2462	0
15	male	25.8521	0
30	male	16.375	6.3764
45	male	10.9901	4.0572
60	male	7.9362	2.5867
70	male	5.2153	1.9021
80	male	2.7979	1.4708
0	female	48.0652	0
5	female	38.8578	0
15	female	30.4496	0
30	female	20.2403	10.4458
45	female	13.1913	7.0091
60	female	8.6265	4.2932
70	female	5.3842	2.7068
80	female	2.8748	1.9002

We do not assume remission from the anginal state in our China model both because rates of revascularization in the stable angina population in China are not well known, and because clinical trials suggest that patients with angina continue to have symptoms after medical or procedural intervention.[12] We specifically state that we assume no remission from angina or heart failure.

Acknowledgements

We would like to acknowledge the contributions of the participants and investigators responsible for the China Multi-provincial Cohort Study, the International Collaborative Study of Cardiovascular Disease in Asia Study, the Sino-MONICA Study, the China National Hypertension Survey Epidemiology Follow-up Study, and the Bridging the Gap in Coronary Heart Disease Secondary Prevention Study, without which this project would not be possible. We would like to thank Dr. Colin Mathers of the Global Burden of Disease Study and the World Health Organization for teaching us the methods and assumptions employed in calculating disability-adjusted life years.

Sources of Funding

Supported by a grant from the Flight Attendants Medical Research Institute, a grant from the Swanson Family Fund to the University of California, San Francisco (to Lee Goldman), and a grant from the William J. Matheson Foundation to Columbia University (to Andrew Moran). These funding bodies had no role in study design, data analysis, data interpretation, writing of the manuscript, or the decision to submit the manuscript for publication.

References

1. **International Database, U.S. Census Bureau.** [http://www.census.gov/ipc/www/idbnew.html]
2. Goodkind DM: **China's missing children: the 2000 census underreporting surprise**. *Population Studies* 2004, **58**(3):281-295.
3. Lozano R, Murray C.J.L., Lopez, A.D., and Satoh, T.: **Miscoding and misclassification of ischaemic heart disease mortality. Global Programme on**

Evidence for Health Policy Working Paper No. 12. . *Geneva, World Health Organization* 2001.

4. He J, Gu D, Wu X, Reynolds K, Duan X, Yao C, Wang J, Chen CS, Chen J, Wildman RP *et al*: **Major causes of death among men and women in China**. *N Engl J Med* 2005, **353**(11):1124-1134.
5. Liu J, Hong Y, D'Agostino RB, Sr., Wu Z, Wang W, Sun J, Wilson PW, Kannel WB, Zhao D: **Predictive value for the Chinese population of the Framingham CHD risk assessment tool compared with the Chinese Multi-Provincial Cohort Study**. *JAMA* 2004, **291**(21):2591-2599.
6. Fischbacher CM, Bhopal R, Unwin N, White M, Alberti KG: **The performance of the Rose angina questionnaire in South Asian and European origin populations: a comparative study in Newcastle, UK**. *International Journal of Epidemiology* 2001, **30**(5):1009-1016.
7. He J, Neal B, Gu D, Suriyawongpaisal P, Xin X, Reynolds R, MacMahon S, Whelton PK: **International collaborative study of cardiovascular disease in Asia: design, rationale, and preliminary results**. *Ethn Dis* 2004, **14**(2):260-268.
8. Garber CE, Carleton RA, Heller GV: **Comparison of "Rose Questionnaire Angina" to exercise thallium scintigraphy: different findings in males and females**. *Journal of Clinical Epidemiology* 1992, **45**(7):715-720.
9. Mathers CD, Truelson, T., Begg, S., and Satoh, T.: **Global burden of ischemic heart diseae in the year 2000. Global Burden of Disease 2000 Working Paper.** *Geneva, World Health Organization* 2004.
10. **Framingham Heart Study CD-ROM: Department of Health and Human Services; 2005.**
11. Tunstall-Pedoe H, Kuulasmaa K, Mahonen M, Tolonen H, Ruokokoski E, Amouyel P: **Contribution of trends in survival and coronary-event rates to changes in**

coronary heart disease mortality: 10-year results from 37 WHO MONICA project populations. Monitoring trends and determinants in cardiovascular disease. *Lancet* 1999, **353**(9164):1547-1557.

12. Weintraub WS, Spertus JA, Kolm P, Maron DJ, Zhang Z, Jurkovitz C, Zhang W, Hartigan PM, Lewis C, Veledar E *et al*: **Effect of PCI on quality of life in patients with stable coronary disease**. *N Engl J Med* 2008, **359**(7):677-687.
